# Supplementary material for: Observation of intrinsic crystal phase in bare few-layer CrI3
Source: Nanophotonics. 2022 Aug 19;11(19):4409–17. doi: 10.1515/nanoph-2022-0246 (PMC11501829; doi:10.1515/nanoph-2022-0246)
Supplement: Supplementary file 1 — Supplementary Material Details [file j_nanoph-2022-0246_suppl.docx]

**Observation of intrinsic crystal phase in bare few-layer CrI_3_**

*Zhen Liu^1,2^, Yongzheng Guo^1,2^, Zhiyong Chen^1,2^, Tao Gong^1,2^, Yue Li^1,2^, Yuting Niu^1,2^, Yingchun Cheng^3^, Haipeng Lu^1,2^, Longjiang Deng^1,2,^* and Bo Peng^1,2,^**

^1^National Engineering Research Center of Electromagnetic Radiation Control Materials, School of Electronic Science and Engineering, University of Electronic Science and Technology of China, Chengdu 611731, China

^2^State Key Laboratory of Electronic Thin Films and Integrated Devices, University of Electronic Science and Technology of China, Chengdu, 611731, China

^3^ Key Laboratory of Flexible Electronics & Institute of Advanced Materials, Jiangsu National Synergetic Innovation Center for Advanced Materials, Nanjing Tech University, Nanjing 211816, China

*Correspondence author’s e-mail: denglj@uestc.edu.cn; bo_peng@uestc.edu.cn

**Table1:** Summary of previous works on low-temperature crystal structure of CrI_3_

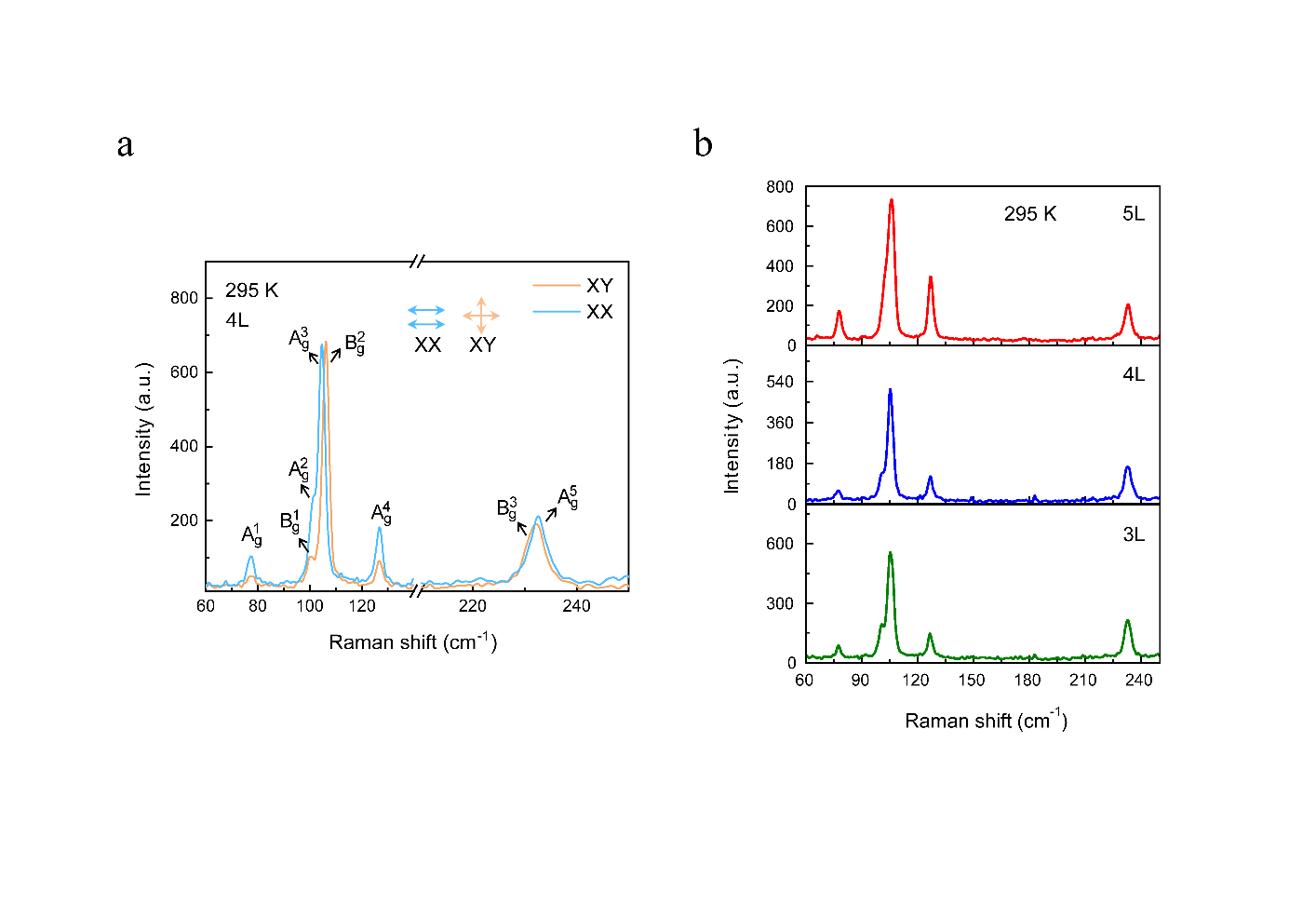


**Figure S1:** Raman spectra of bare 3-5L CrI_3_. (a) Raman spectrum of bare 4L CrI_3_ collected in the linearly parallel (XX) and crossed (XY) polarization channels at 295 K. (b) Non-polarized Raman spectra of bare 3-5L CrI_3_ at 295 K.


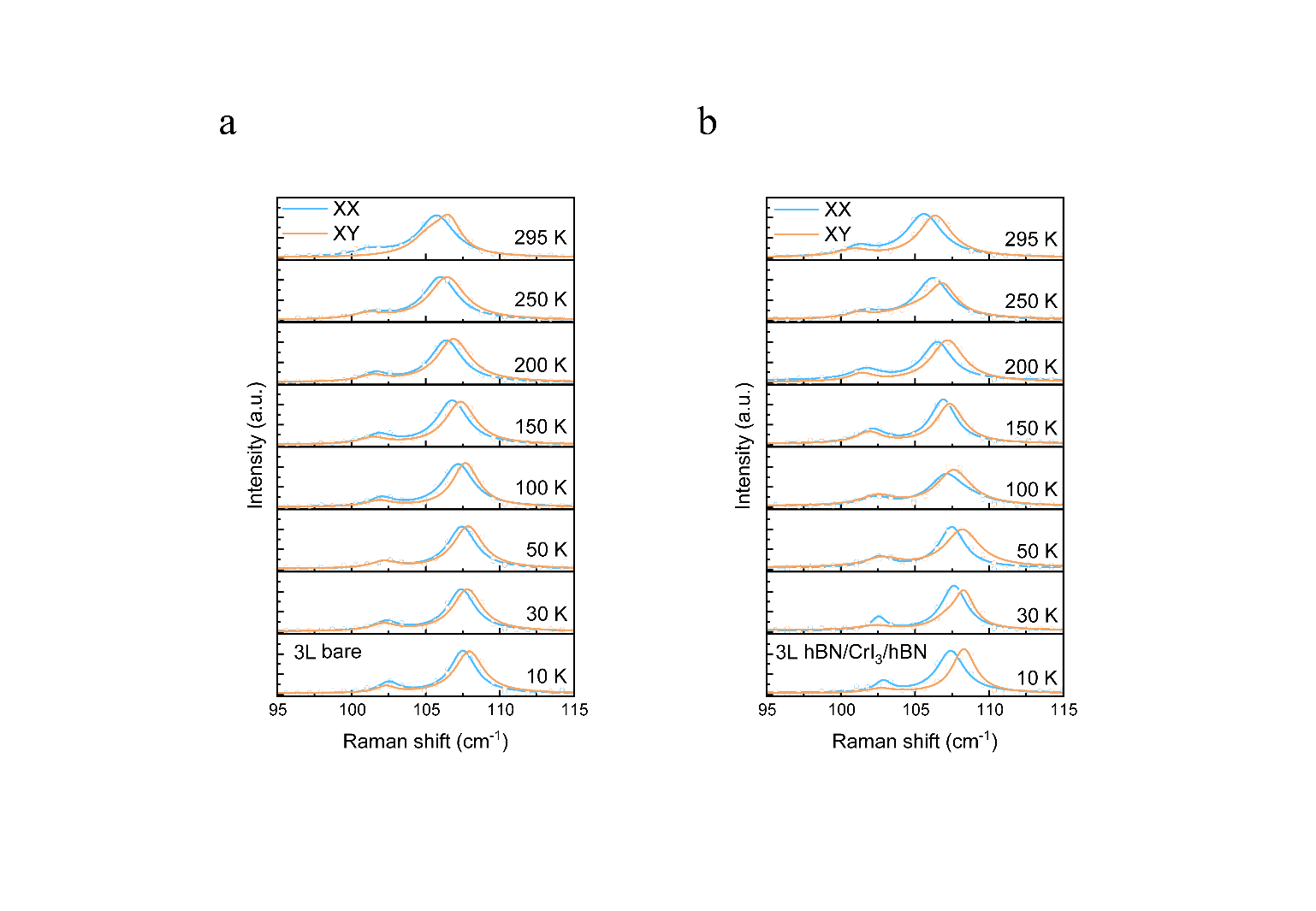


**Figure S2:** hBN-encapsulation hindering interlayer lateral sliding. (a)-(b) Temperature-dependence Raman spectra of bare and hBN-encapsulated 3L CrI_3_ in the linearly parallel (XX) and crossed (XY) polarization channels, respectively.


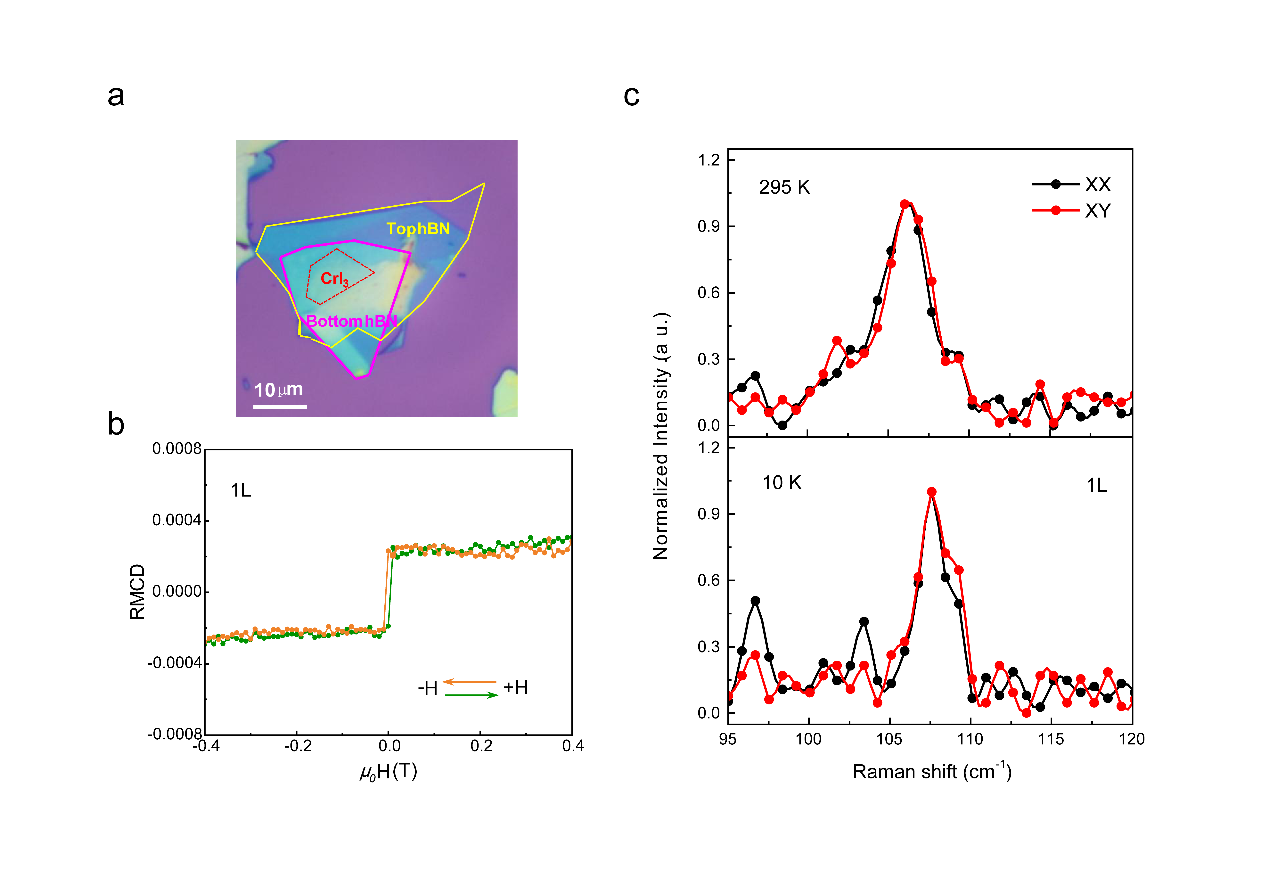


**Figure S3:** Raman and RMCD measurements on monolayer CrI_3_. (a) Optical image of hBN-encapsulation 1L CrI_3_. The scale bar is 10 μm. (b) RMCD spectrum of 1L CrI_3_ taken at 10 K. (c) Raman spectra of 1L CrI_3_ in the linearly parallel (XX) and crossed (XY) polarization channels, respectively.


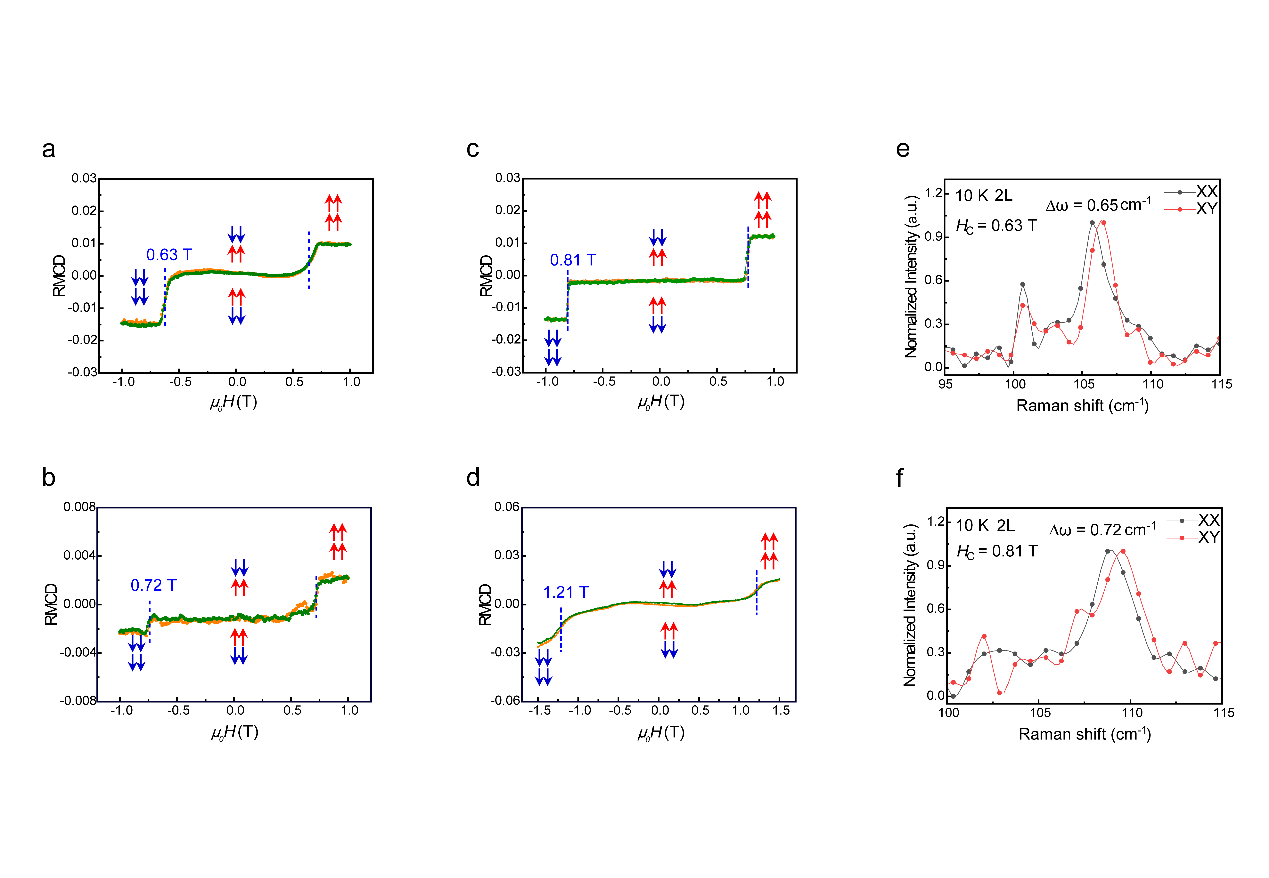


**Figure S4:** RMCD and Raman measurements on different four 2L CrI_3_ samples. (a)-(d) RMCD spectra of four different 2L CrI_3_ samples taken at 10 K. (e)-(f) Dual-channel Raman signals of two different 2L CrI_3_ taken at 10 K.


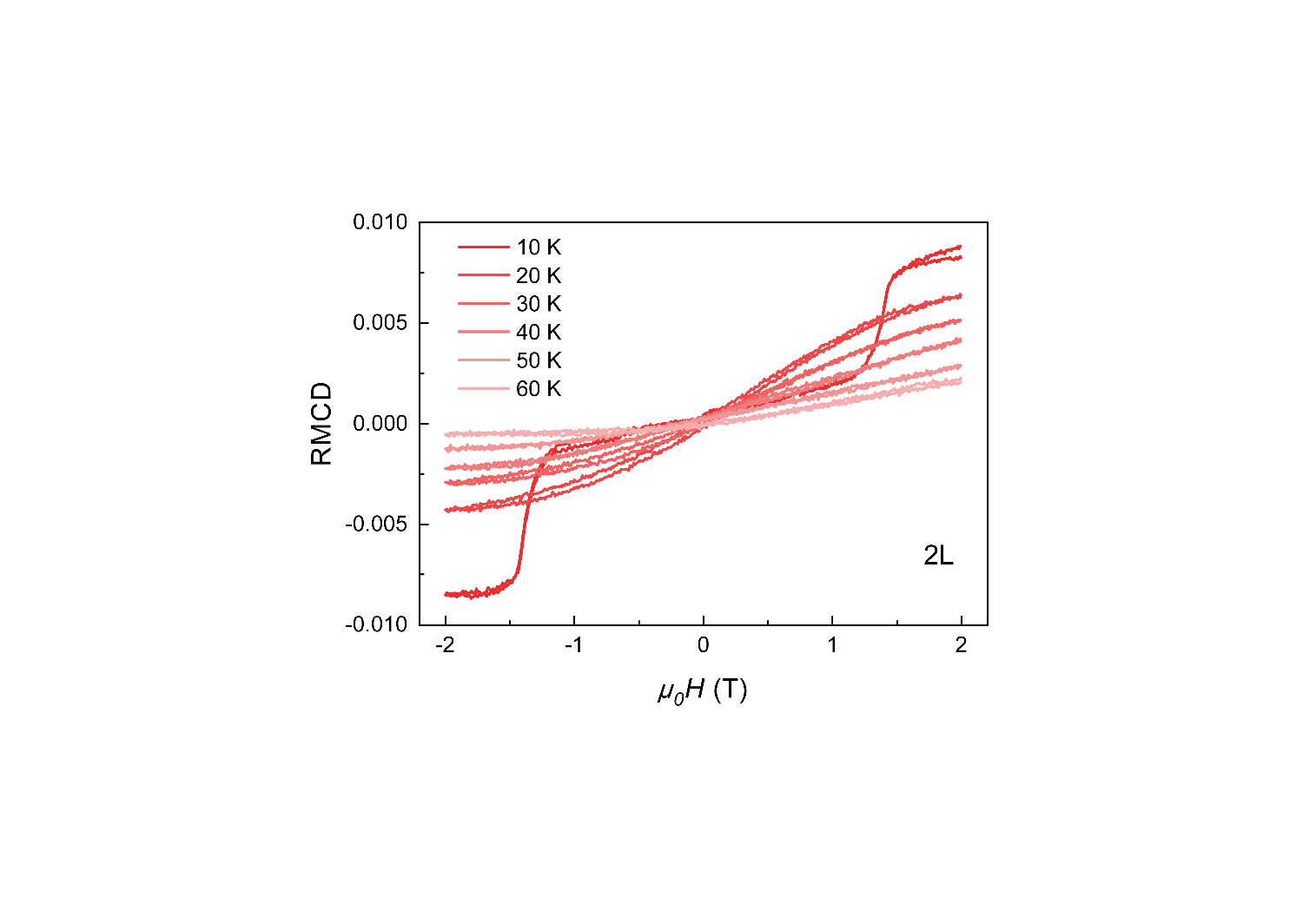


**Figure S5:** Temperature-dependence RMCD spectra of bare 2L CrI_3_ from 10 to 60 K.


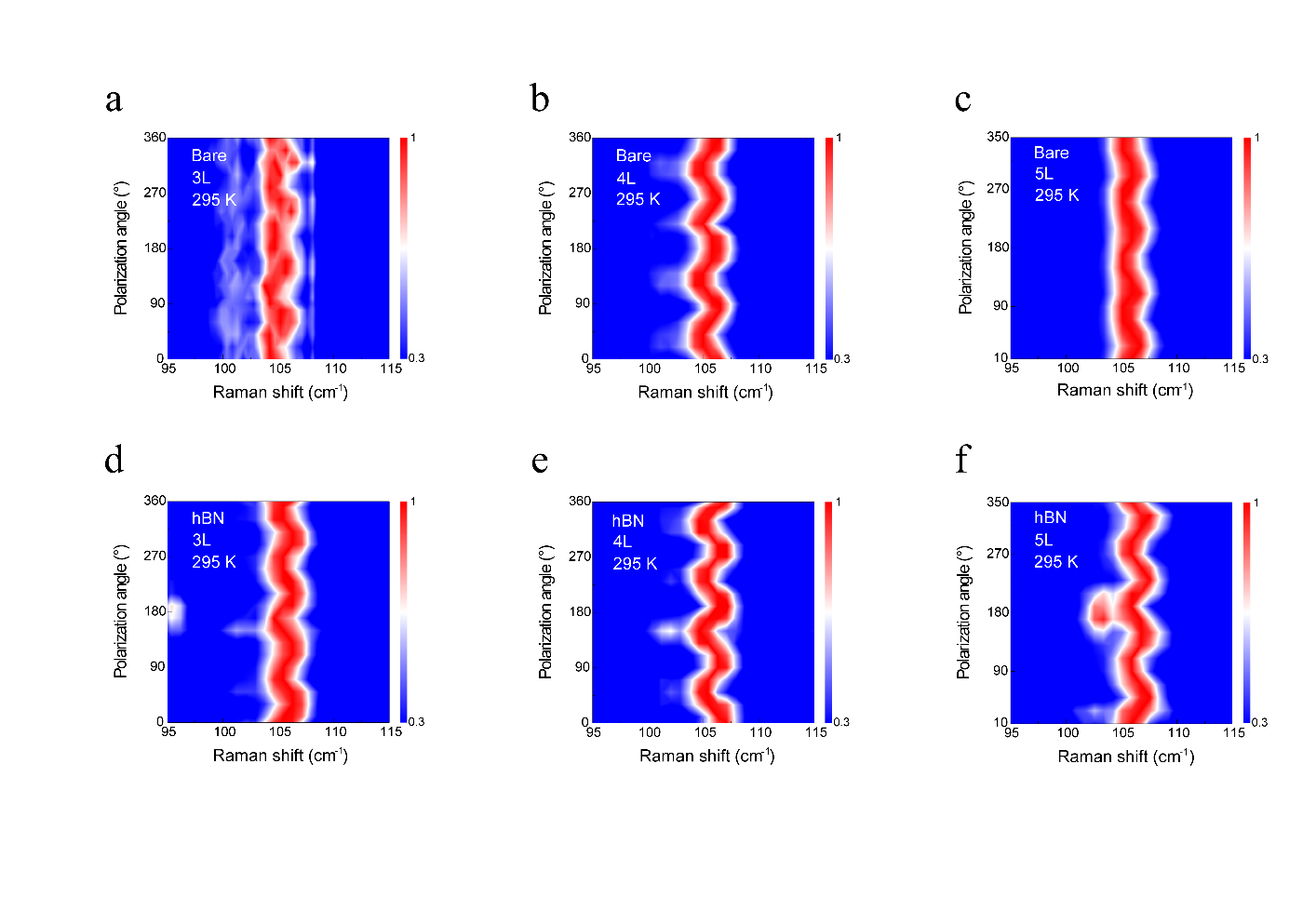


**Figure S6:** Intrinsic monoclinic phase of bare and encapsulated 3-5L CrI_3_ at 295 K. (a)-(c) Polarization angle dependence of Raman spectra of bare 3-5L CrI_3_ at 295 K. (d)-(f) Polarization angle dependence of Raman spectra of hBN-encapsulated 3-5L CrI_3_ at 295 K.


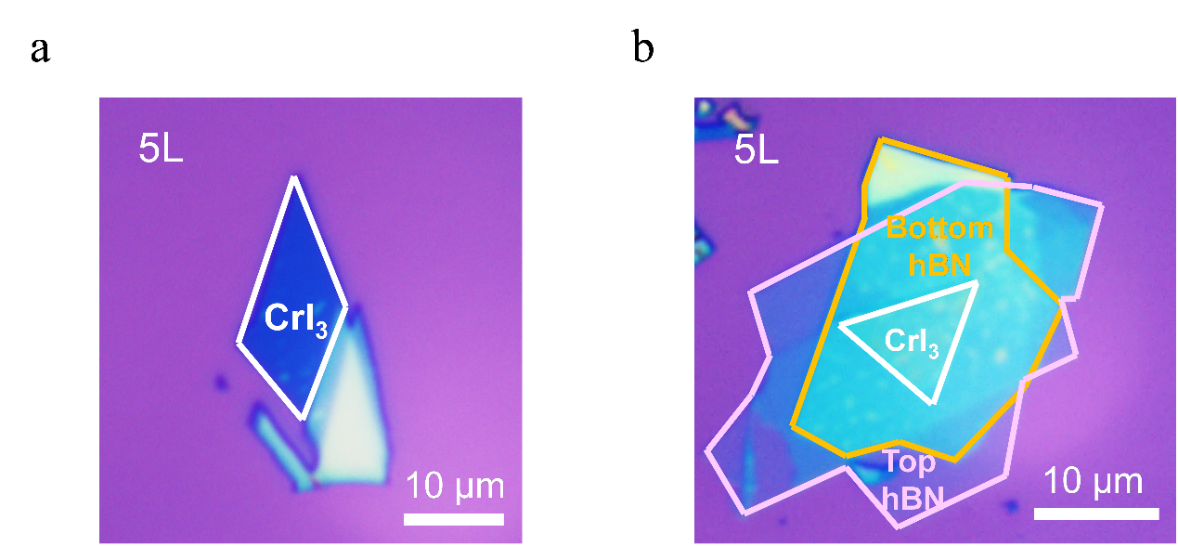


**Figure S7:** Comparison of optical images of 5L CrI_3_. (a) bare 5L CrI_3_. (b) hBN-encapsulated 5L CrI_3_.


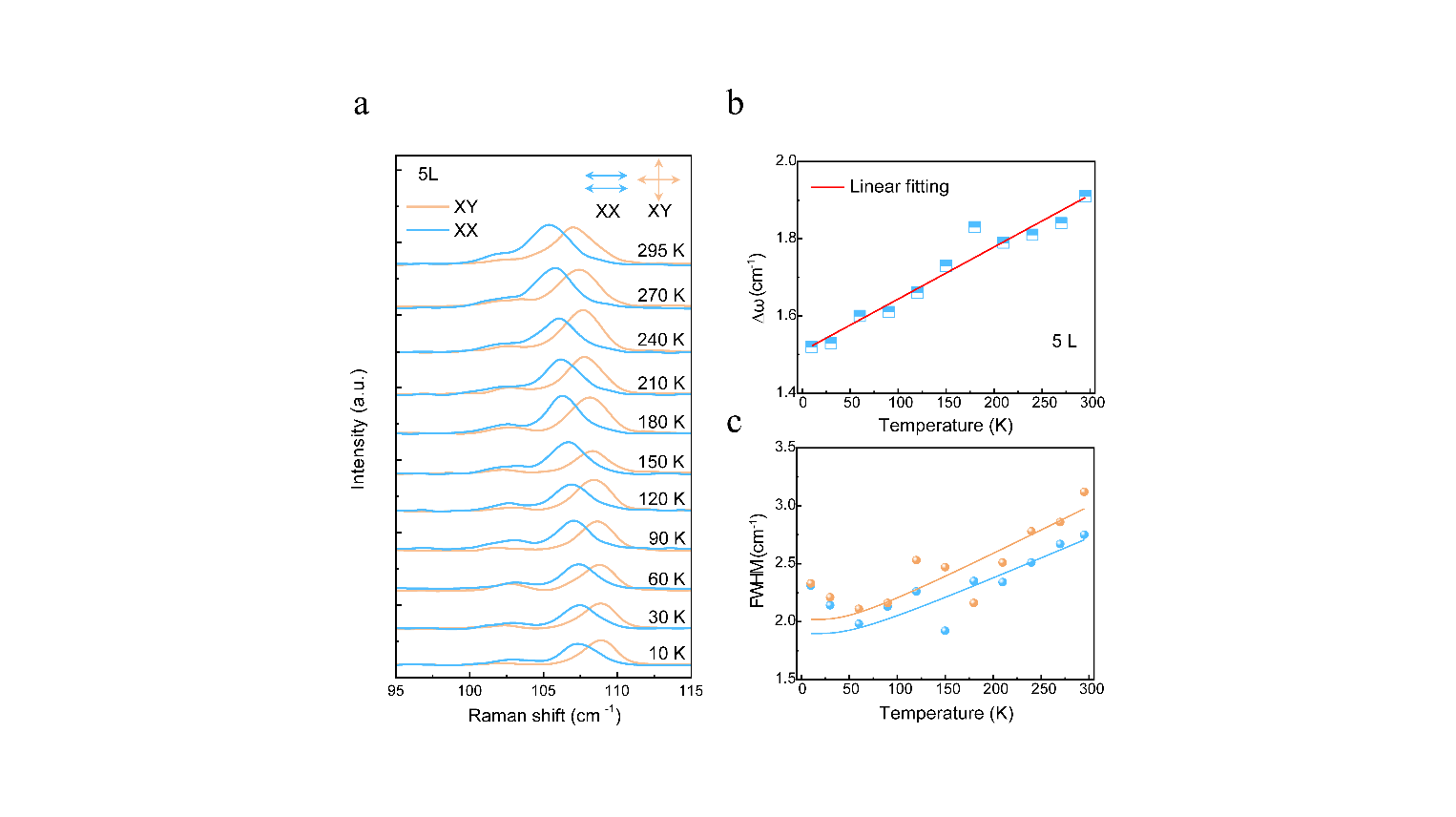


**Figure S8:** Monoclinic stacking of bare 5L CrI_3_ with temperature. (a) Temperature-dependence Raman spectra of bare 5L CrI_3_ collected in the linearly parallel (XX) and crossed (XY) polarization channels. Spectra are vertically offset for clarity. (b) The energy difference of $\text{A}_{\text{g}}$ and $\text{B}_{\text{g}}$ modes at ~107 cm^-1^ as a function of temperature extracted from linearly parallel (XX) and crossed (XY) polarization Raman spectra in Figure. S6(a). Monoclinic stacking is always preserved with decreasing temperature and no phase transition takes place. (c) The linewidths of the $\text{A}_{\text{g}}$ and $\text{B}_{\text{g}}$ modes as a function of temperature extracted from Figure. S6(a). The sudden increase in linewidth indicates the occurrence of spin-lattice coupling and the transition temperature corresponds to the Curie temperature of 5L CrI_3_.

**
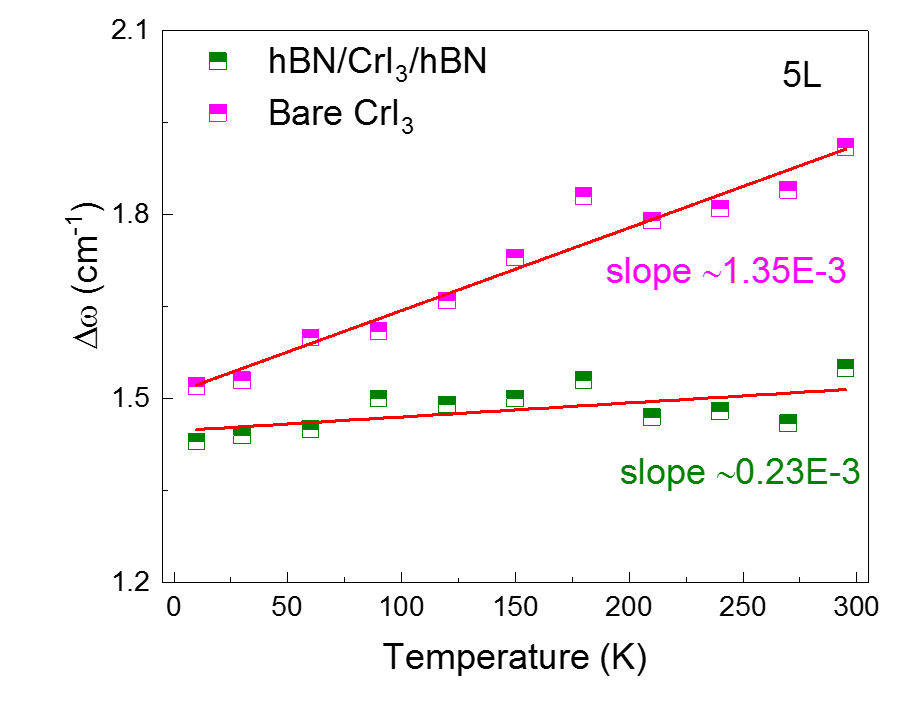
**

**Figure S9:** The Raman mode energy difference as a function of temperature in separate bare and hBN-encapsulated 5L CrI_3_. The extracted slope are 1.35E-3 and 0.23E-3 for the fully-bare and fully-encapsulated samples, respectively.


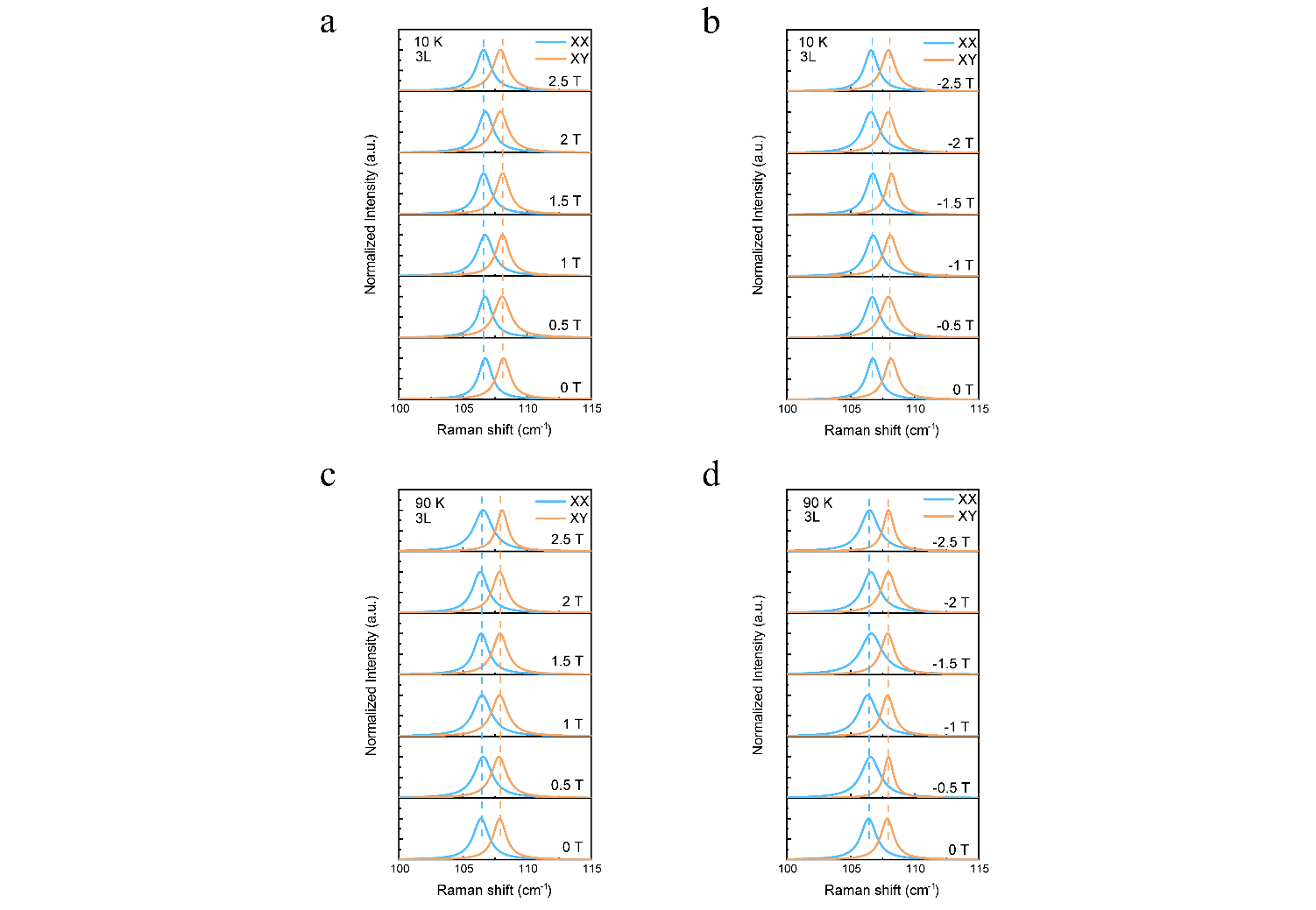


**Figure S10:** Magnetic-dependence Raman spectra of bare 3L CrI_3_. (a)-(b) Two split modes in the linearly parallel and crossed channels as a function of selected positive (negative) out-of-plane magnetic fields at 10 K. (c)-(d) Two split modes in the linearly parallel and crossed channels as a function of selected positive (negative) out-of-plane magnetic fields at 90 K. The Raman modes are fitted by Lorenz function. Two split modes of bare 3L CrI_3_ are independence on magnetic field.


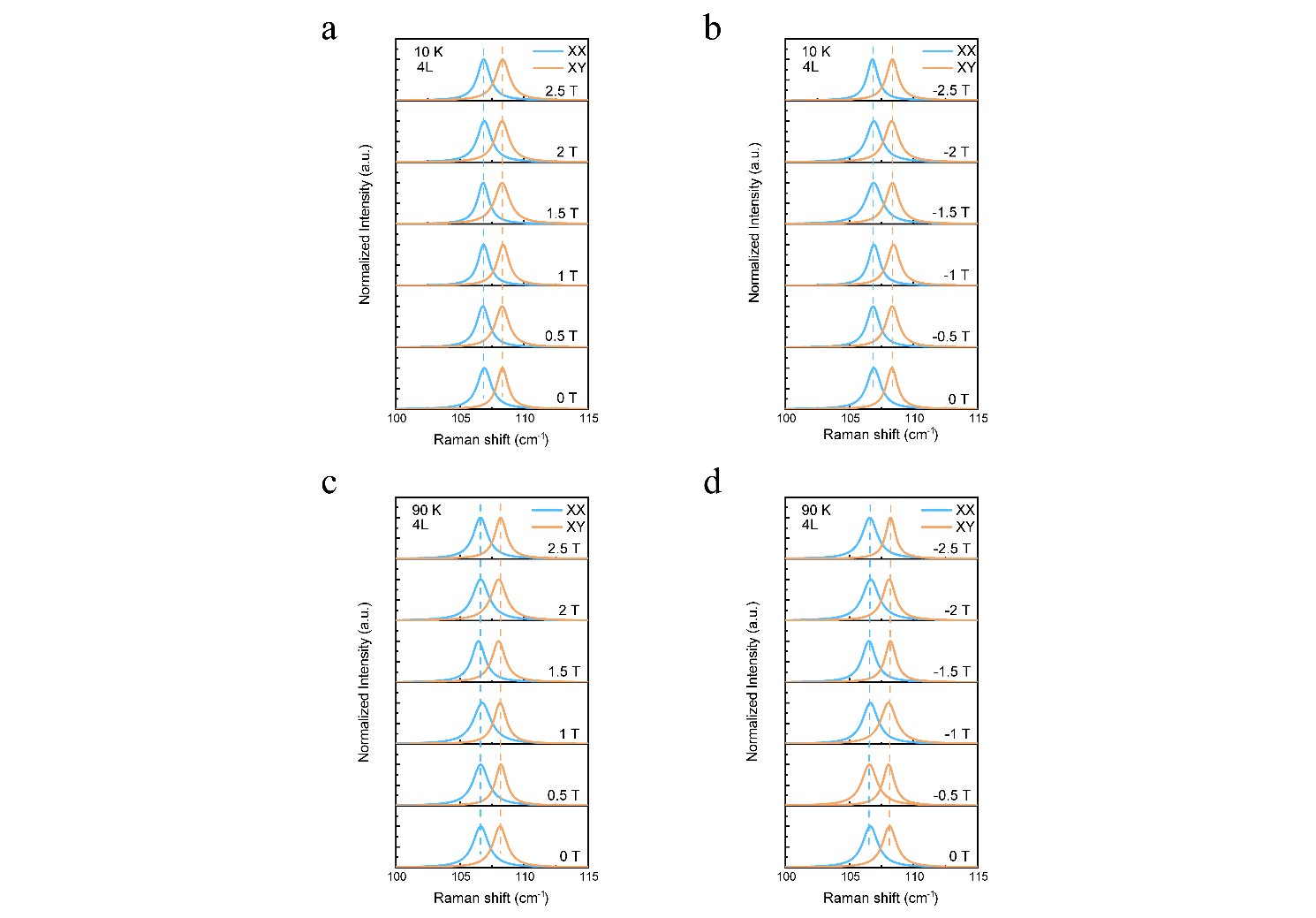


**Figure S11:** Magnetic-dependence Raman spectra of bare 4L CrI_3_. (a)-(b) Two split modes in the linearly parallel and crossed channels as a function of selected positive (negative) out-of-plane magnetic fields at 10 K. (c)-(d) Two split modes in the linearly parallel and crossed channels as a function of selected positive (negative) out-of-plane magnetic fields at 90 K. The Raman modes are fitted by Lorenz function. Two split modes of bare 4L CrI_3_ are independence on magnetic field.


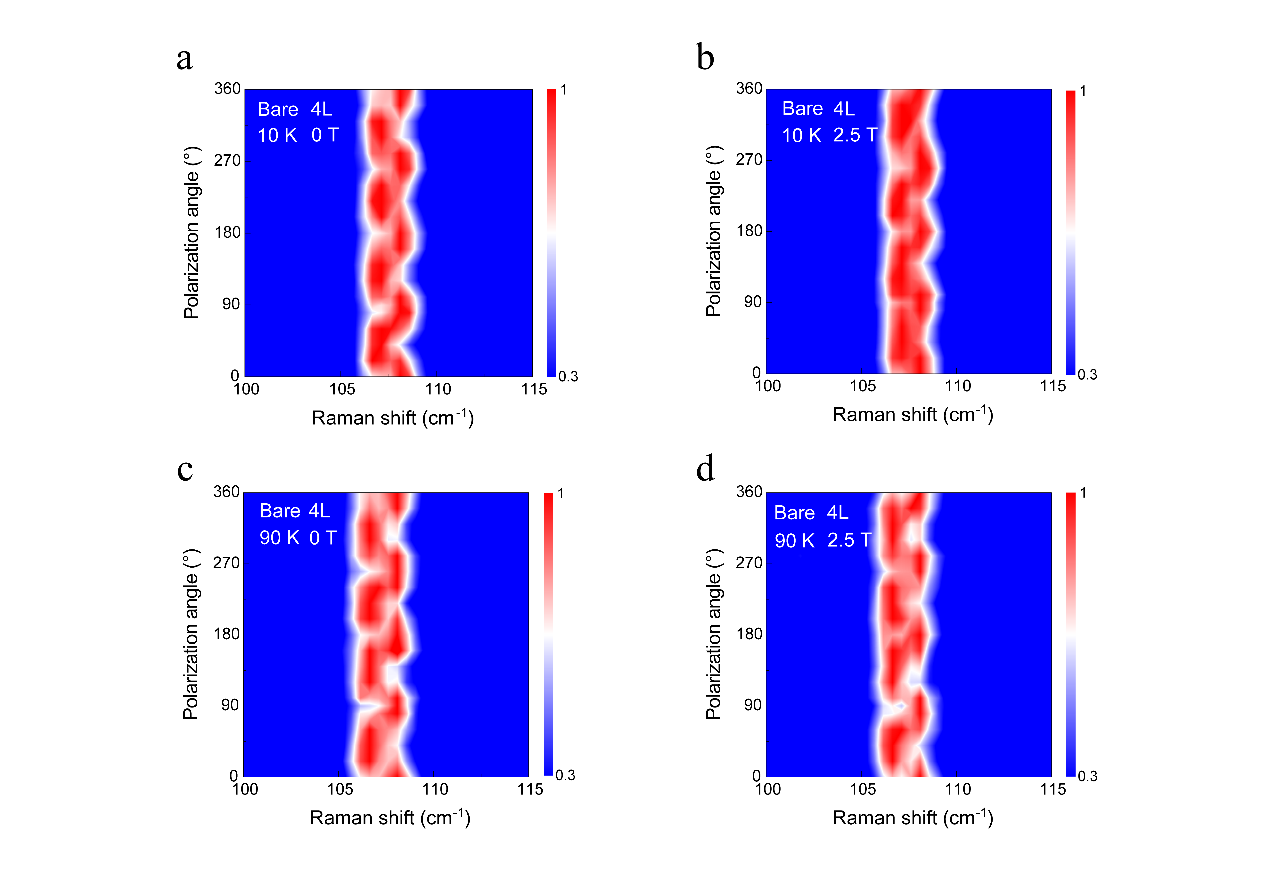


**Figure S12:** Magnetic-field independence of monoclinic phase of bare 4L CrI_3_. (a)-(b) Polarization angle dependence of Raman spectra of bare 4L CrI_3_ collected at 0 and 2.5 T at 10 K. (c)-(d) Polarization angle dependence of Raman spectra of 4L CrI_3_ collected at 0 and 2.5 T at 90 K.
